# Traumatic Ulcerative Granuloma With Stromal Eosinophilia (TUGSE): A Case Report

**DOI:** 10.7759/cureus.71579

**Published:** 2024-10-16

**Authors:** Deeksheetha Prabhu Venkatesh, Karthikeyan Ramalingam, Pratibha Ramani, Rajprakash Bhaskaran

**Affiliations:** 1 Oral Pathology and Microbiology, Saveetha Dental College and Hospitals, Saveetha Institute of Medical and Technical Sciences, Saveetha University, Chennai, IND; 2 Oral and Maxillofacial Surgery, Saveetha Dental College and Hospitals, Saveetha Institute of Medical and Technical Sciences, Saveetha University, Chennai, IND

**Keywords:** chronic, dental trauma, early diagnosis, eosinophilia, granuloma, inflammation, sharp tooth, tongue, trauma, ulcer

## Abstract

Traumatic ulcerative granuloma with stromal eosinophilia (TUGSE) is a rare, benign lesion often misdiagnosed due to its clinical and histological similarities with malignant conditions. It typically presents as a persistent, non-healing ulcer in the oral cavity. This case report describes a 64-year-old female patient who presented with a solitary ulcer caused by chronic trauma from a supra-erupted tooth. Histopathological findings of an ulcerated lesion with intense inflammation, comprising eosinophils, confirmed the diagnosis of TUGSE. The offending cusp was treated, and the ulcer healed without any complications. This case report enforces prompt recognition and removal of the traumatic source, which will lead to healing, emphasizing the importance of early diagnosis and intervention in TUGSE.

## Introduction

Traumatic ulcerative granuloma with stromal eosinophilia (TUGSE) is a rare, benign, yet persistent oral lesion that frequently mimics malignancy, making it a diagnostic challenge. It commonly presents as a non-healing, solitary ulcer that arises in response to trauma [1]. Clinically, it presents as a slow-healing ulcer with elevated or rolled borders, closely resembling squamous cell carcinoma, with a healing period ranging from one week to a year [2,3]. The base of the ulcer may be yellowish to white, with a surrounding erythematous border. There is no gender predilection reported in previous studies, and the lesion resolves on its own without any intervention and seldom recurs [4]. The condition is marked by a prominent infiltration of eosinophils deep into the connective tissues, leading to significant tissue damage. TUGSE has a preference for the tongue and buccal mucosa, as these areas are prone to repeated trauma from teeth or dental prostheses; however, it can also occur in other oral mucosal sites, such as the palatal mucosa, floor of the mouth, gingiva, vestibular area, and retromolar trigone area [5].

Histopathologically, the hallmark of TUGSE is an intense inflammatory infiltrate, particularly eosinophils, which extend deep into the connective tissue stroma, as well as the underlying skeletal muscle and minor salivary glands [2]. The overlying ulcerated epithelium shows pseudoepitheliomatous hyperplasia. The histopathological features can resemble those of squamous cell carcinoma. A lesion with similar histopathological features, most commonly seen on the ventral surface of the tongue of infants and children, is Riga-Fede disease, which occurs due to trauma from the developing mandibular incisors [4,5].

TUGSE was first described by Elzay in 1983. It was further studied by Ficarra and Kaban in 1997, who emphasized its association with local trauma [1]. It usually appears as a single lesion that raises suspicion of malignancy. Histopathology shows granulation tissue with ulcerated areas and dense inflammation containing eosinophils. It usually heals spontaneously within a month. Various reports of TUGSE have underscored its rarity and diagnostic complexity [1-6].

In this case, a 64-year-old woman presented with a solitary, well-defined ulcer caused by trauma from the supra-erupted cusp of a mandibular molar. This report aims to expand on the clinical, histological, and therapeutic aspects of TUGSE while comparing this case with previous studies to highlight the diagnostic challenges and management strategies associated with this rare lesion.

## Case presentation

A 64-year-old female presented to the Outpatient Department of Saveetha Dental College and Hospitals, Chennai, India, with a tongue ulcer that had been present for the past four weeks. The ulcer had been present for four weeks without healing, pain, or discomfort during mastication. Her past medical history, surgical history, and dental history were non-contributory.

**Figure 1:**
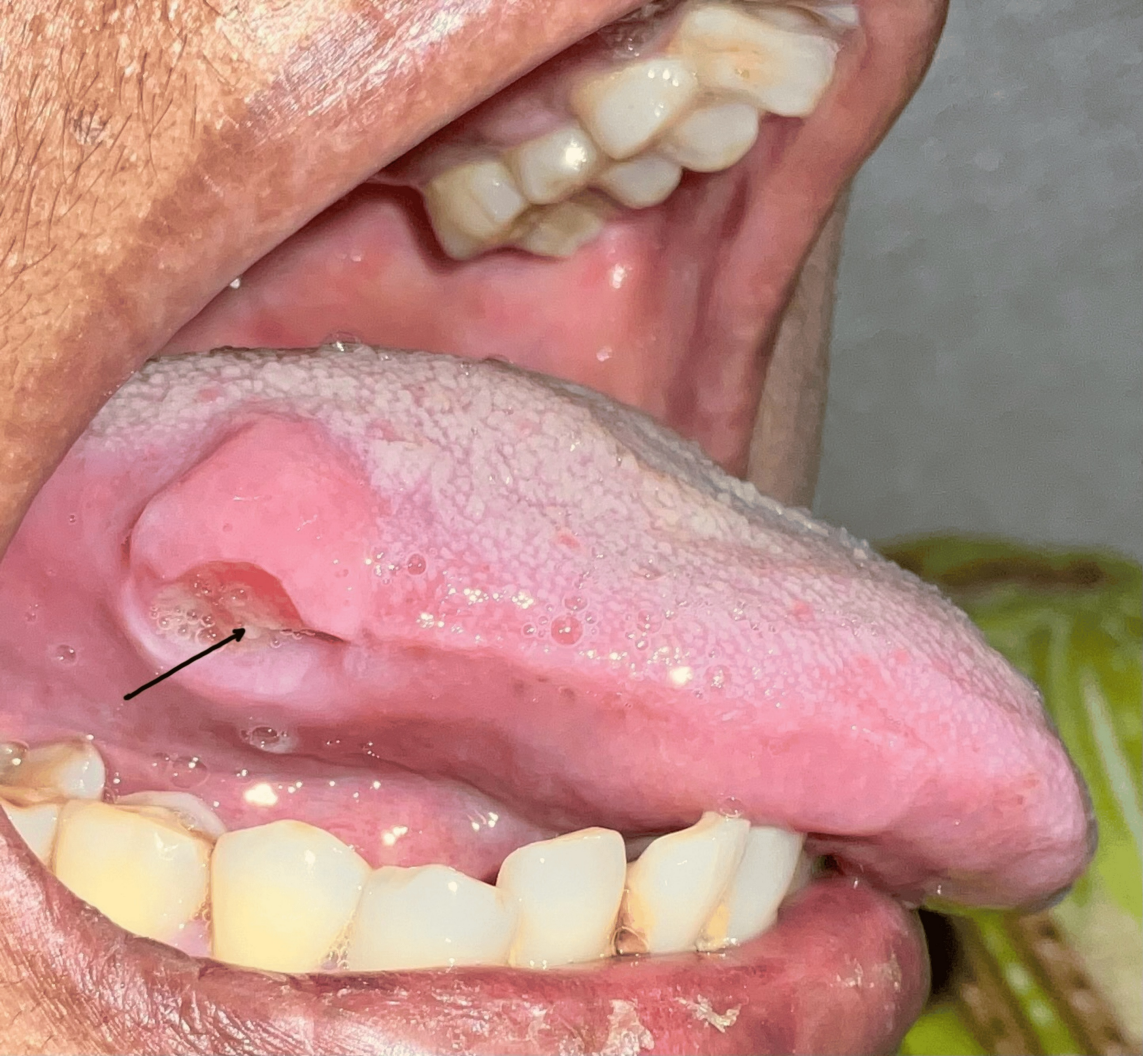
Clinical image The clinical image reveals a single, well-defined ulcer with raised edges on the right lateral border of the tongue. The black arrow shows the ulcerated lesion.

An informed consent was obtained from the patient for further examination and diagnosis. There were no abnormalities on the extraoral examination. Intraoral examination revealed a well-defined ulcer measuring approximately 2 x 2 cm in size. An indurated base and tenderness were elicited on palpation. The ulcer was located on the right lateral border of the tongue, adjacent to the supra-erupted mandibular first molar (46), which appeared to be impinging on the ulcerated area (Figure 1).

**Figure 2:**
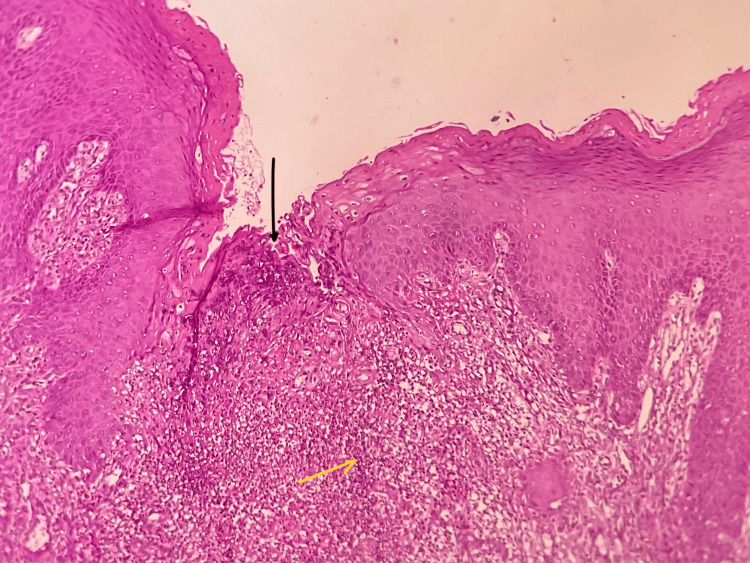
Photomicrograph H&E-stained sections show ulcerated, parakeratinized stratified squamous epithelium with ulceration (black arrow). The yellow arrow shows an intense inflammatory cell infiltrate extending deep into the connective tissue stroma (H&E, 20x). H&E: hematoxylin and eosin

Other oral findings were unremarkable. Due to the chronic and indurated nature of the lesion, a provisional diagnosis of traumatic ulcer was made, and an incisional biopsy was performed to rule out any malignancy. Multiple hematoxylin and eosin (H&E) stained sections were examined. The sections revealed a large area of ulceration surrounded by hyperparakeratinized stratified squamous epithelium of variable thickness, with features of pseudoepitheliomatous hyperplasia. Beneath the epithelium, there was an intense mixed inflammatory infiltrate predominantly composed of eosinophils, along with lymphocytes, neutrophils, plasma cells, and numerous blood vessels. The eosinophils were seen infiltrating deep into the underlying fibrous connective tissue and skeletal muscle, a hallmark of TUGSE. Moderate vascularity and areas of hemorrhage were noted within the lesion, along with the presence of adipose tissue and nerve bundles. These findings were consistent with TUGSE, and no malignant cells were identified (Figure 2).

**Figure 3:**
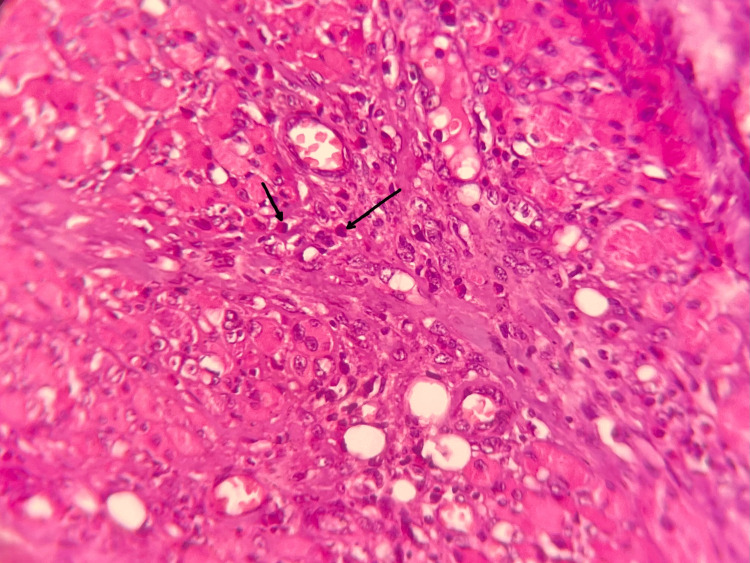
Photomicrograph H&E-stained sections show an intense inflammatory cell infiltrate with numerous eosinophils (black arrows) (H&E, 40x). H&E: hematoxylin and eosin

Evaluation of the inflammatory cell infiltrate revealed numerous eosinophils in the connective tissue stroma (Figure 3).

The offending cusp of the mandibular molar (46) was rounded by coronoplasty. The patient was advised to use a topical anesthetic gel, avoid spicy food, and maintain regular oral hygiene. The patient reported satisfactory healing and alleviation of pain and discomfort in the tongue over the follow-up period of six weeks. The patient did not return to the clinic after the suggested treatment to record photographs. Feedback from the voice call confirmed that the lesion had healed and she was asymptomatic.

## Discussion

TUGSE is an uncommon, self-limiting condition that often arises due to mechanical trauma, such as impingement from teeth or dental appliances [2]. TUGSE is most commonly seen in the lateral borders of the tongue [4]. In this case, the supra-erupted cusp of tooth 46 was identified as the source of chronic irritation, contributing to the development of the ulcer. The indurated base and slow healing process raised suspicion for malignancy, a common challenge in diagnosing TUGSE.

Our clinical presentation was characterized by a solitary ulcer with indurated margins and tenderness on palpation; these findings were consistent with the previous case reports [7,8]. Meeral and Arumugham [9] reported that ulcers were the chief complaint in only 2.97% of the cases. TUGSE is an even rarer condition within this small percentage. Similar presentations in their cohort of TUGSE cases, where trauma from sharp tooth edges or dental appliances was frequently the initiating factor for TUGSE, were reported [1,3]. In our patient, the supra-erupted mandibular molar was identified as the causative agent. This is consistent with studies by Shen et al. [2], who found that in over 80% of cases, the source of trauma could be attributed to dental factors, such as sharp cusps or ill-fitting prosthetics.

Histologically, the hallmark feature of TUGSE is the dense eosinophilic infiltrate that extends into the deep connective tissues and skeletal muscle. In our case, eosinophils were abundant in the ulcerated area, penetrating deep into the fibrous connective tissue and skeletal muscle, confirming the diagnosis [2,3]. This is consistent with Mohamad et al. [6], who highlighted the importance of eosinophils in the pathogenesis of TUGSE, suggesting that they may play a role in tissue destruction and repair through the release of cytokines and enzymes. Immunohistochemical analysis revealed that the inflammatory cells were CD3 and CD68 positive [6].

While the exact pathogenesis of TUGSE remains unclear, eosinophils play a central role in the lesion's development. Their presence, along with other inflammatory cells, suggests an immune-mediated response to chronic trauma [2,4]. Eosinophils contribute to the chronic inflammatory process by releasing granules containing cytotoxic proteins, enzymes, and cytokines. This may explain the tissue necrosis and damage observed in these ulcers [7,10]. It has been suggested that eosinophils are not merely bystanders but active participants in the pathogenesis of TUGSE [6,7]. Elovic et al. [8] noted that the delayed healing of oral TUGSE lesions may be linked to the insufficient production of transforming growth factor (TGF)-α and TGF-β by eosinophils. Their infiltration into muscle tissues suggests a robust immune response, which may be triggered by persistent trauma [2]. The findings in our case, including the intense eosinophilic infiltrate and the extent of tissue involvement, further support the hypothesis of an eosinophil-mediated process.

One of the major challenges in diagnosing TUGSE is its resemblance to malignancies, such as squamous cell carcinoma, or other chronic ulcerative conditions, like infections or autoimmune diseases [11]. The indurated base and chronicity of the ulcer raised clinical concern for malignancy, warranting a biopsy [4]. This mirrors the experience of other clinicians, where TUGSE is often diagnosed only after histopathological examination due to its deceptive clinical appearance [3-8]. Ficarra et al. [11] reported that many patients with TUGSE underwent multiple diagnostic tests due to concerns about oral cancer before arriving at a definitive diagnosis. The presence of ulcerated epithelium with pseudoepitheliomatous hyperplasia, as seen in our case, complicates the diagnosis by mimicking squamous cell carcinoma to the untrained eye.

Multiple studies reported complete resolution of TUGSE lesions following the elimination of the traumatic insult [1-8]. Malignant lesions, like oral squamous cell carcinoma, do not show healing and persist after eliminating local causes [12]. Post-operative follow-up is difficult in such cases, as our patient did not report after the resolution of the tongue lesion. Post-treatment records are not available in these case reports in PUBMED [13,14]. TUGSE resolves once the local trauma is mitigated, underscoring the importance of addressing the underlying cause. Although the tongue is the primary organ for detecting taste sensations [15], it is also the most susceptible to trauma [2]. While there are no specific diagnostic criteria for TUGSE based on clinical appearance alone, eosinophils penetrating deep tissues remain a key histological finding [14]. Lakkam et al. [13] emphasized that the deep infiltration of eosinophils into muscle and the surrounding connective tissue is pathognomonic of TUGSE and helps differentiate it from other ulcerative conditions [13]. In our case, the deep penetration of eosinophils into the muscle tissue was evident, corroborating the findings of previous studies. Thomas et al. [16] have discussed chronic mechanical irritation leading to oral ulcers. Novel therapeutic combinations are being used for their healing properties [17].

TUGSE has an excellent prognosis once the traumatic etiology is addressed, and recurrence is uncommon. In our case, the prompt recognition and elimination of the irritant led to the complete healing of the lesion, underscoring the importance of early intervention. We have summarized the findings from the reported literature about TUGSE in Table 1.

**Table 1:** Summary of reported literature on TUGSE TUGSE: traumatic ulcerative granuloma with stromal eosinophilia; M: male; F: female

Author	Age and gender	Clinical features	Histopathological features	Treatment
SahanaPushpa and Balamurugan (2022) [1]	45/F	Pain in the left lateral border of the tongue for the past 8 months	Ulcerated epithelium with granulomatous connective tissue stroma with intense inflammation	Surgical excision
Banerjee et al. (2021) [3]	35/F	Solitary ulcer right buccal mucosa with carious upper molar (25)	Dense granular lesion with numerous inflammatory cell infiltrate	Extraction of 26, with regular follow-up; follow up for 3 months
Kacar et al. (2024) [5]	63/M	Indurated ulcer on the dorsum of the tongue	Classical features of TUGSE	The lesion healed after an incisional biopsy
Mohamad et al. (2023) [6]	13/M	Painful solitary indurated ulcer in the lower anterior vestibule	Ulcerated epithelium with fibrous connective tissue stroma with intense inflammatory infiltrate with numerous eosinophils	The lesion healed after an incisional biopsy
Benitez et al. (2019) [10]	48/M	Single ulcer in lower left retromolar trigone region	A granulomatous lesion with dense inflammatory cell infiltrate with numerous eosinophils	Review and follow-up for 26 months; no recurrence of the lesion; lesion healed uneventfully after incision and extraction of 37
Lakkam et al. (2021) [13]	65/M	The ulcer was seen on the right lateral border of the tongue, which was small in size and increased gradually in size	Ulcerated fibrinopurulent membrane with dense inflammatory cell infiltrate in the connective tissue stroma along with numerous eosinophils	Surgical excision of the lesion was done
Hannan et al. (2020) [18]	21/M	Solitary white plaque in the left palatoglossal region	A granulomatous lesion with dense inflammatory cell infiltrate with numerous eosinophils	The lesion healed uneventfully after an incisional biopsy
Cheng and Laura (2017) [19]	6/M	Non-healing ulcer in right buccal mucosa	Classic features of TUGSE	Healed after incision biopsy; no recurrence of the lesion during the 6-week follow-up
Shokravi et al. (2022) [20]	51/F	Ulcer associated with pain in the soft palate	Ulcerated fibrinopurulent membrane with dense inflammatory cell infiltrate in the connective tissue stroma along with numerous eosinophils	Lesion healed uneventfully after incisional biopsy; follow-up for 3 months
Krishna et al. (2023) [21]	52/M	Pain and ulcer in the left buccal mucosa for the last 20 days; the presence of root stump in relation to 26	Features of TUGSE	Resolved after extraction of 26
Sidana et al. (2023) [22]	27/M	Ulcer in left posterior buccal mucosa	Features compatible with TUGSE	The ulcer disappeared completely after a month
Azori et al. (2023) [23]	13/F	The ulcer was seen on the ventral surface of the tongue; associated with the removal appliance	Features compatible with TUGSE	Appliance use was discontinued and excision of the lesion was done; no recurrence of the lesion
Tanasubsinn (2021) [24]	67/F	Large painful chronic ulcer with indurated and irregular surface left buccal mucosa; the patient had a history of several recurrent episodes in one and a half years	A large zone of surface ulceration consisting of a thin fibrinopurulent membrane; there was the presence of dense lymphohistiocytic and mononuclear cell chronic inflammatory infiltrate with numerous eosinophils extending into the fibrous connective tissue below the ulcer	Ulcer resolved spontaneously after tissue biopsy; the possible etiological factor supra-erupted 26 was removed; no recurrence was found after a 6-month follow-up
Muñoz and Ulloa (2021) [25]	56/F	An ovoid pedunculated lesion with a smooth surface that had a tendency to bleed	Beneath an atrophic, parakeratinized stratified squamous epithelium, there was a proliferation of fibroblastic tissue with abundant dilated vessels and neoformation, featuring foci of hemorrhage and a mixed inflammatory infiltrate comprising histiocytes, lymphocytes, plasmocytes, and abundant eosinophils	The patient did not have any recurrence after the excisional biopsy

## Conclusions

TUGSE should be considered in the differential diagnosis of non-healing oral ulcers, particularly when there is a history of trauma. This case highlights the importance of recognizing TUGSE as a distinct clinical entity, despite its similarity to malignancies and other ulcerative conditions. Traumatic injury to the tongue from the sharp tooth is a key diagnostic feature in our case, and histopathological confirmation of the ulcerated lesion, granulation tissue, and inflammation with eosinophils was essential for the diagnosis. Early identification and removal of the traumatic source lead to rapid resolution and prevent unnecessary diagnostic procedures or treatments.
